# Development of an Imaging Spectrometer with a High Signal-to-Noise Ratio Based on High Energy Transmission Efficiency for Soil Organic Matter Detection

**DOI:** 10.3390/s24134385

**Published:** 2024-07-05

**Authors:** Jize Fan, Yuwei Wang, Guochao Gu, Zhe Li, Xiaoxu Wang, Hanshuang Li, Bo Li, Denghui Hu

**Affiliations:** 1 Changchun Institute of Optics, Fine Mechanics and Physics, Chinese Academy of Sciences, Changchun 130033, China; fanjize20@mails.ucas.ac.cn (J.F.);; 2 University of Chinese Academy of Sciences, Beijing 100049, China; 3 Innovation Academy for Microsatellites, Chinese Academy of Sciences, Shanghai 200100, China

**Keywords:** hyperspectral, optical design, remote sensing, signal-to-noise ratio, spectrometer

## Abstract

Hyperspectral detection of the change rate of organic matter content in agricultural remote sensing requires a high signal-to-noise ratio (SNR). However, due to the large number and efficiency limitation of the components, it is difficult to improve the SNR. This study uses high-efficiency convex grating with a diffraction efficiency exceeding 50% across the 360–850 nm range, a back-illuminated Complementary Metal Oxide Semiconductor (CMOS) detector with a 95% efficiency in peak wavelength, and silver-coated mirrors to develop an imaging spectrometer for detecting soil organic matter (SOM). The designed system meets the spectral resolution of 10 nm in the 360–850 nm range and achieves a swath of 100 km and a spatial resolution of 100 m at an orbital height of 648.2 km. This study also uses the basic structure of Offner with fewer components in the design and sets the mirrors of the Offner structure to have the same sphere, which can achieve the rapid adjustment of the co-standard. This study performs a theoretical analysis of the developed Offner imaging spectrometer based on the classical Rowland circular structure, with a 21.8 mm slit length; simulates its capacity for suppressing the +2nd-order diffraction stray light with the filter; and analyzes the imaging quality after meeting the tolerance requirements, which is combined with the surface shape characteristics of the high-efficiency grating. After this test, the grating has a diffraction efficiency above 50%, and the silver-coated mirrors have a reflection value above 95% on average. Finally, the laboratory tests show that the SNR over the waveband exceeds 300 and reaches 800 at 550 nm, which is higher than some current instruments in orbit for soil observation. The proposed imaging spectrometer has a spectral resolution of 10 nm, and its modulation transfer function (MTF) is greater than 0.23 at the Nyquist frequency, making it suitable for remote sensing observation of SOM change rate. The manufacture of such a high-efficiency broadband grating and the development of the proposed instrument with high energy transmission efficiency can provide a feasible technical solution for observing faint targets with a high SNR.

## 1. Introduction

A higher SNR is required for the accurate detection of SOM in black soil with a low surface reflectance and a low annual variation rate. This method can accurately distinguish between background noise and variation rate signals. The use of satellite data with different ground resolutions and SNRs for SOM detection has shown that the observation instrument needs a wide swath and a high SNR [1,2,3,4,5]. The area of black soil distribution in the world is approximately 100 to 200 square kilometers. Currently, a substantial number of research studies on soil organic matter are based on the convenient collection of remote sensing data, with the most widely used payloads being land satellites, MODIS, and Sentinel satellites. Using these data, it has been found that in large areas of fertile black soil between 40° and 45° north latitude, the planting window is concentrated around May, with a solar elevation angle generally around 30°, and the surface albedo is approximately 30%. Thus, the observation instrument needs an SNR that is higher than 300 in the 360–850 nm range. Meanwhile, to avoid snow and crop coverage, the observation window needs to be set within 15 days, which has the characteristic of a short period; therefore, the observation instrument needs to have a swath width of 100 km [6]. Higher SOM content results in darker soil with lower reflectance, thus necessitating an improved signal-to-noise ratio. Additionally, VIS/NIR spectroscopy has been proven to be a reliable tool for predicting the pH value and OM content of unknown soil samples [7,8]. Observing SOM with a spectral resolution of 10 nm in the visible–near-infrared (VNIR) region can achieve an accuracy approximately that of 1 nm, with reduced data volume [9,10]. At the same time, a spectrometer with a high SNR can also be designed with key components for traceability according to the International System of Units for calibration. In such a transfer link, the imaging spectrometer system must meet the requirements of high SNRs and low stray light signals [11,12].

The SNR of remoting sensing instruments for soil observation can be improved in some ways. Firstly, the detectors used can combine time-delay integration technology with back-illumination technology. When a detector is in the binning mode, since the number of photo-generated electrons is related to the pixel area, increasing the pixel area by a multiplier can directly increase the number of photo-generated electrons of the target to improve the SNR due to the merging of pixels. Furthermore, because the full well capacity is further expanded in the binning mode, the upper limit of the SNR can also be increased without exposure [13,14,15,16]. The other way is using a prism instead of grating to improve the efficiency of dispersion components [17]. In the binning mode, Gaofen-5 (GF-5) uses an Offner spectrometer with a meniscus to achieve an SNR of 250 in the VNIR region, with a swath of 60 km. The Environmental Monitoring and Analysis Program (EnMAP)’s VNIR channel employs a curved prism dispersion method to achieve an SNR of 100–300, but its swath is only 30 km. The Hyper-spectral Imager SUIte (HISUI)’s VNIR channel merges four pixels in the spectral dimension, achieving an SNR of 450 at 620 nm, but its swath is only 20 km. The F numbers of all of the above-mentioned on-orbit instruments are around three, and they still face the issue of insufficient swath in the detection of SOM. In the latest research, the Offner imaging spectrometer achieves a swath width of 400 km (0.64°) in stationary orbit, but its F number is only 6.75 in the VNIR range. Meanwhile, the grating efficiency reaches 50–80% in the 300–550 nm range and 50–74% in the 550–1010 nm range. Therefore, there is still room for improvement in diffraction efficiency over a broader bandwidth [18]. Considering the superior linear dispersion capability of gratings compared to prisms, we ultimately selected gratings as the dispersion element.

To improve the SNR of a spectrometer, the F number of the optical system can also be reduced to increase the incident energy. However, reducing the F number will increase the numerical aperture (NA), leading to a large angle of incidence for edge rays and, thus, causing greater aberrations. Optical systems may require complex surface shapes or additional components to adapt to changes in the F number, such as a highly aspherical or freeform surface [19,20,21,22,23]. The ELOIS uses two highly aspherical mirrors and a free-form diffraction grating to achieve an F number of 2.1 and an SNR of 150–500 in the VNIR channel. The EIS mission uses a free-form surface grating to achieve an F number of 1.8 and an SNR of 150–400 within the 400–2450 nm range. In a recent fluorescence detection study, a combination of aberration correction lens groups, prisms, and volume-phase holographic grating was employed to achieve imaging quality with an F number of 2. Although the F number was very small, the final SNR only reached 200.

Some instruments mentioned above are based on Dyson-type designs, which are more suitable for instruments with small F numbers. In contrast, due to their larger off-axis amount caused by the slit, it is challenging to design Offner systems with a small F number. Generally, an F number ranging from 2.5 to 3 can be relatively small for Offner structures. However, smaller F numbers imply more severe aberrations, necessitating the use of complex surfaces or the introduction of additional optical elements such as corrective lenses. Increasing the number of components can lower the throughput, thereby affecting the SNR. In this case, reducing the F number will synchronize with the decrease in efficiency, resulting in no significant change in the SNR. The development of grating imaging spectrometers with a high SNR requires a high grating efficiency in the wide waveband range, a high quantum efficiency for the detector in the shortwave range, and a superior imaging quality of the optical system with a low F number.

## 2. Theory and Methods

### 2.1. SNR of Slit Imaging Spectrometer with CMOS

**Figure 1:**
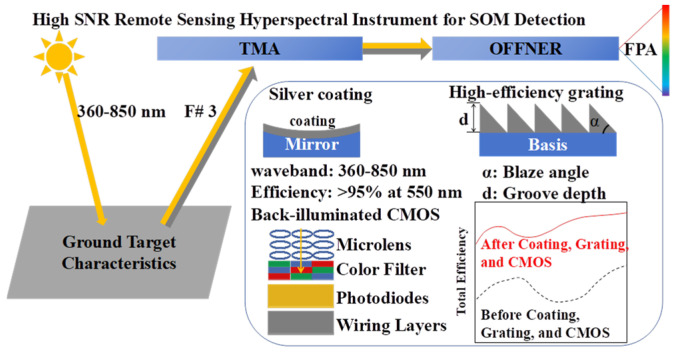
The method of improving the SNR of a hyperspectral instrument for SOM detection.

The number of photo-generated electrons of the detector from which the irradiance of the ground reflection signal is analyzed is calculated as follows:

(1) $$N_{signal}=\frac{\pi \cdot \Phi \cdot A_{0}\cdot t\cdot T\left(\lambda \right)\cdot \eta \left(\lambda \right)}{4h\cdot c\cdot F^{2}}L\left(\lambda \right)\cdot \lambda \cdot \Delta \lambda$$

where *L* represents the reflected irradiance of the ground surface, *T* is the transmission efficiency of the optical system, *h* is the Planck constant, *c* is the speed of light, *F* is the F number of the optical system, *λ* is the wavelength, *Φ* is the fill factor of the detector, *A*_0_ is the pixel area, *t* is the integration time, *η* is the quantum efficiency of the detector, and Δ*λ* is the spectral sampling rate of a single pixel. The SNR calculation equation under the condition of applying a CMOS detector is given as follows [24]:

(2) $$SNR=\frac{N_{singal}}{\sqrt{N_{singal}+N_{dark}+\sigma_{read}^{2}}}=\frac{\frac{\pi \cdot \Phi \cdot A_{0}\cdot t\cdot T\left(\lambda \right)\cdot \eta \left(\lambda \right)}{4h\cdot c\cdot F^{2}}L\left(\lambda \right)\cdot \lambda \cdot \Delta \lambda }{\sqrt{\frac{\pi \cdot \Phi \cdot A_{0}\cdot t\cdot T\left(\lambda \right)\cdot \eta \left(\lambda \right)}{4h\cdot c\cdot F^{2}}L\left(\lambda \right)\cdot \lambda \cdot \Delta \lambda +N_{dark}+\sigma_{read}^{2}}}$$

where $N_{dark}$ represents the number of dark current electrons and $\sigma_{read}$ represents the readout noise. Based on this equation, the SNR can be effectively enhanced by minimizing the detector noise; increasing the full well capacity, which will raise the theoretical upper limit of the SNR; increasing the energy transmission capacity of the grating and mirrors; and using a faster F number. This study used silver-coated mirrors, a back-illuminated CMOS detector, and high-efficiency grating technologies and chooses the Tilted Mirror Anastigmat (TMA) and Offner structures to minimize the number of optical surfaces, as shown in Figure 1. The detection of SOM targets is based on the reflection information from the surface in the 360–850 nm range, which is obtained using spectral imaging through the TMA and Offner systems with fewer components. There is a focal-plane array (FPA) to receive the image. This setup incorporates silver-coated mirror technology, achieving a peak reflectance of 95% at 550 nm. Additionally, the grating’s blaze angle and groove depth are specially designed to maintain an efficiency above 50% across the wavelength range. Using thinned back-illuminated CMOS technology further enhances the detector’s quantum efficiency, particularly providing higher response levels at shorter wavelengths. By integrating these technologies, the overall efficiency of the instrument is improved, thereby enhancing the SNR level.

### 2.2. Offner Spectrometer Based on Rowland Circle

**Figure 2:**
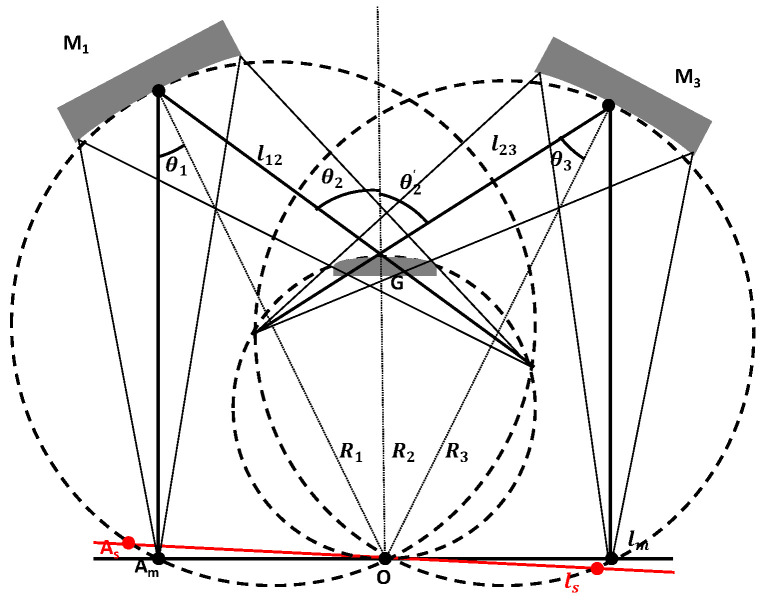
Rowland circle in the meridional plane.

The Offner spectrometer originated from the Rowland circular fundamental structure with minimal aberrations. It particularly focuses on the imaging point separation between the meridional and sagittal planes, called astigmatism. This section describes an astigmatism analysis conducted based on the Rowland circle to search for the minimum astigmatism at the central wavelength as the initial parameters for designing curvatures and distances. Furthermore, the dispersion length is analyzed by calculating the incident angle, radius, and groove of grating to obtain the initial structure. The optical path is shown in Figure 2.

In this figure, M_1_ represents the first mirror of the Offner system, with a curvature radius of $R_{1}$. G represents the grating inside the Offner system, with a curvature radius of $R_{2}$. M_3_ represents the third mirror of the Offner system, with a curvature radius of $R_{3}$. $\theta_{n}$ denotes the angle of incidence of the chief ray relative to the nth surface. $l_{12}$ is the optical path of the incidence point between M_1_ and G, and $l_{23}$ is the optical path between G and M_3_. $l_{m}$ and $l_{s}$ are the imaging distances in the meridional and sagittal planes, respectively.

The radius of the convex grating and spectral dispersion length can be written as follows:

(3) $$R_{2}=\frac{L_{spec}}{mn\Delta \lambda }$$

where *m* is the selected diffraction of the +1st order. After confirmation with the manufacturer, the initial range of the grating groove is selected to be around 60 lines/mm. $L_{spec}$ can be obtained from the formula $L_{spec}>2.2\;\mathrm{mm}>2\times pixel\;size\times \Delta \lambda /10$, where Δλ is the wavelength in the 360–850 nm range (490 nm), and the pixel size is 22 μm. The grating radius $R_{2}$ is calculated as 74.83 mm. The incidence angle of grating can be written as follows:

(4) $$\theta_{2}\approx \arcsin\left\{nm\bar{\lambda }+\sin\left[\arctan\left(\frac{1}{F}+\frac{h_{spec}+s}{R_{2}}\right)\right]\right\}$$

where F is the F number, which is 3; $\bar{\lambda }$ is the central wavelength, which is 605 nm; s is the redundant distance between the grating edge and the side light, which is 10 mm; and the initial value of $\theta_{2}$ is 28.76°. According to the sine theorem and the distribution of right triangles, the following equations can be obtained:

(5) $$R_{1}\sin\theta_{1}=R_{2}\sin\theta_{2}$$

(6) $$l_{12}=\frac{R_{1}\sin\left(\theta_{2}-\theta_{1}\right)}{\sin\theta_{2}}$$

Combining the grating equation and Equations (3)–(6), the expression corresponding to $l_{m}$ can be written as follows:

(7) $$\left\{\begin{array}{l} \sin\theta_{2}^{\prime }=\sin\theta_{2}-mn\lambda \\ \sin\theta_{3}=\frac{R_{2}\sin\theta_{2}^{\prime }}{R_{3}}\\ l_{23}=\frac{R_{3}\sin\left(\theta_{2}^{\prime }-\theta_{3}\right)}{\sin\theta_{2}^{\prime }}\\ l_{m}=R_{3}\cos\theta_{3} \end{array}\right.$$

Combining the relationship of the sagittal and meridional planes, the formulas for calculating the distances and radii in the sagittal plane are as follows:

(8) $$\left\{\begin{array}{c} \frac{1}{R_{1}\cos\theta_{1}}+\frac{1}{l_{s1}^{\prime }}=\frac{2\cos\theta_{1}}{R_{1}}\\ \frac{1}{l_{s1}^{\prime }-l_{12}}+\frac{1}{l_{s2}^{\prime }}=\frac{\cos\theta_{2}+\cos\theta_{2}^{\prime }}{R_{2}}\\ \frac{1}{l_{s2}^{\prime }+l_{23}}+\frac{1}{l_{s}}=\frac{2\cos\theta_{3}}{R_{3}} \end{array}\right.,$$

where $l_{s1}^{\prime }$, $l_{s2}^{\prime }$, and $l_{s}$ represent, respectively, the image distance for each component in the sagittal direction, the final imaging distance, and the sagittal image point, which can be expressed as

(9) $$l_{s}=1/\left(\frac{2\cos\theta_{3}}{R_{3}}-\frac{1}{l_{s2}^{\prime }+l_{23}}\right)$$

When there is no astigmatism in the system, it means that the sagittal and meridional image points coincide, and the distance between them is 0. This can be expressed by the following equation:

(10) $$\left|l_{s}-l_{m}\right|=0$$

Due to the short distance, the scalar form is used instead of the $l_{s}$ and $l_{m}$ vector (x, y) form to approximately calculate the initial parameters of the structure. When confirming the initial structure of the Offner configuration, the primary focus is on calculating the curvature radius of each component. The input conditions for this calculation include the instrument’s required dispersion length $L_{spec}$, the chosen diffraction order (m) for the grating (which is +1st order in this case), and the spectral range of 360–850 nm. Utilizing these parameters, the curvature radius $R_{2}$ of the grating can be determined. Next, Equations (7)–(9) are used to solve for the positions of the sagittal and meridional image points at the central wavelength $\bar{\lambda }$. Finally, Formula (10) is employed, where at the central wavelength and at the center of the field of view, the distance between two image points is zero when there is no astigmatism. This process ultimately confirms the curvature radii required for the two mirrors. The resulting initial structure yields the following curvature radii: the curvature radius of the first mirror is 118.4 mm, the curvature radius of the grating is 74.8299 mm, and the curvature radius of the third mirror is 120.1 mm. These values are crucial for establishing the optimal configuration of the Offner spectrometer, ensuring its effective performance within the specified spectral range.

## 3. Design and Analyses

### 3.1. Optical Design

The instrument’s orbital height is fixed at 648.2 km, the swath width is 100 km, the ground resolution (GSD) is 100 m, the spectral resolution is 10 nm, and the F number is 3. Based on the general formulas for remote sensing instruments, parameters such as focal length, field-of-view (FOV) angle, and slit length can be calculated.

The focal length is determined using the following formula:

(11) $$f=\frac{a\times H}{GSD}$$

where f is the focal length, a is the pixel size, and H is the orbital height. The calculated system focal length is 141.42 mm. Since the Offner system has a magnification ratio of 1, the telescope’s focal length is also 141.42 mm.

The FOV angle can be obtained using the following formula:

(12) FOV=arctan(L/2H)

where L is the swath, and the calculated field-of-view angle that meets the requirement is ±4.41°. The slit length can be calculated using the following formula:

(13) slitlength=2×tan(FOV)×f=21.8mm

The entrance pupil size can be expressed as follows:

(14) D=f/Fnumber=47.14mm

The above parameters are listed in Table 1.

**Figure 3:**
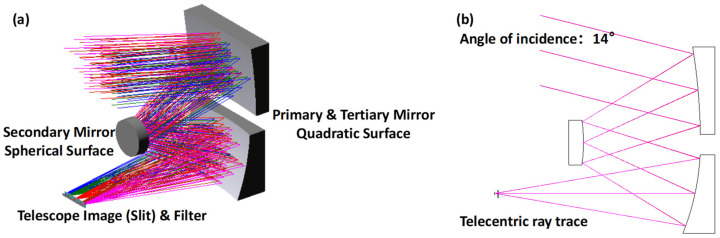
Telescope’s optical structure (**a**); ray trace of TMA (**b**).

A TMA system was selected for its non-chromatic aberration and good performance in imaging aberrations. In the design process, considering the co-alignment of various component positions, there is no eccentricity or tilt set for the three mirrors relative to the stop position (secondary mirror). This ensures that the centers of curvature of the three mirrors are coaxial, and the axial distances from the stop to the other two mirrors are equal. Considering that using the TMA system, after constraining the relative positions of the components, may result in a degradation of imaging capability, different quadratic concave mirrors with different k (conic constant of the surface) values were used to counteract the decrease in imaging quality. The ray trace is shown in Figure 3; there is a filter placed in front of the imaging plane, which is used to select the working waveband and block the ash. In addition to laboratory testing, there is a need to observe soil during flight tests, and the aircraft will inhale a large amount of dust during takeoff and landing. To prevent dust from blocking the slit, the filters placed on both sides of the slit not only select the working wavelength range but also serve to protect the slit.

**Figure 4:**
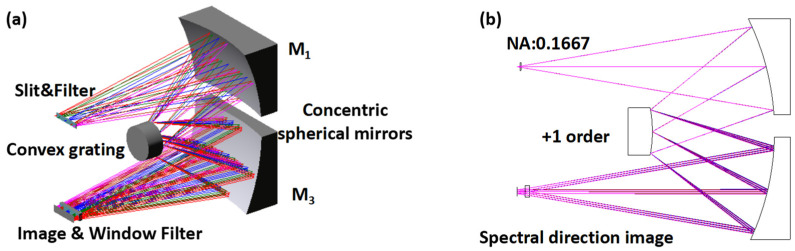
Spectrometer’s optical structure (**a**) and ray trace (**b**).

After the above calculation, the length of the slit is 21.8 mm and the F number is 3. By combining the radii and optical path distances calculated in Section 2 with the grating groove and grating incident angle, this study proceeded to design an object-side telecentric imaging spectrometer. To obtain a common standard of assembly for the two reflective surfaces in the Offner structure, a design with the same spherical parameters for both mirrors was used. During the manufacturing process, the use of this design allowed for the fabrication of a single spherical surface, which was then cut to achieve the required effective diameter. During assembly, the two reflecting mirrors were treated as a single surface, allowing relative positioning using surface testing methods. To meet the imaging quality requirements, the parameters of the grating surface were optimized. The resulting ray trace is shown in Figure 4. At the position of the grating, there were distances greater than 10 mm left on both the top and bottom for grating clamping. Before the imaging plane, a parallel plane was placed to simulate the imaging effect of a window filter.

**Figure 5:**
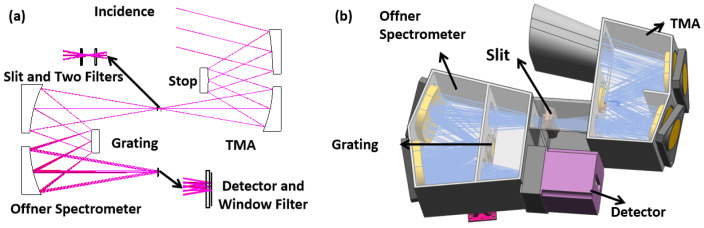
Optical system’s ray trace (**a**) and structure (**b**).

The optical structure of the whole instrument is mainly composed of an Offner imaging spectrometer and a TMA structure. The two parts are connected at the slit. Figure 5 shows the design results of the optical system for the whole instrument. The structure size is 90 mm × 250 mm × 340 mm.

The parameters of the surfaces and distances of the spectrometer are listed in Table 2.

In this table, “component” represents each surface, “Radius” denotes the radius of curvature of each surface, “Thickness” indicates the distance from a surface to the next adjacent surface, and “k” is the conic constant of the surface.

### 3.2. Optical Imaging Quality and Tolerance

**Figure 6:**
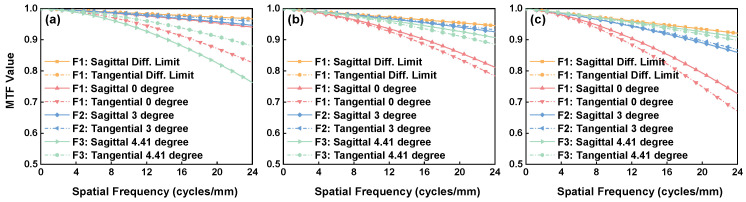
The MTF curves of the optical structure: 360 nm (**a**); 600 nm (**b**); and 850 nm (**c**).

**Figure 7:**
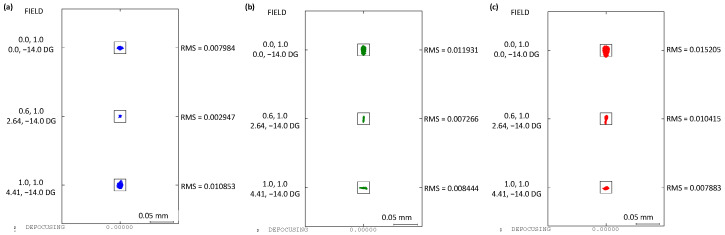
The RMS of the optical structure: 360 nm (**a**); 600 nm (**b**); and 850 nm (**c**).

The imaging quality of the whole structure is shown in Figure 6 and Figure 7. At different wavelengths, the meridional and sagittal MTF curves for various field points are all above 0.7 at the 22.7 lp/mm cut-off frequency. The Root Mean Square (RMS) diagram shows a radius less than 15 μm, with the box representing the size of the pixels.

**Figure 8:**
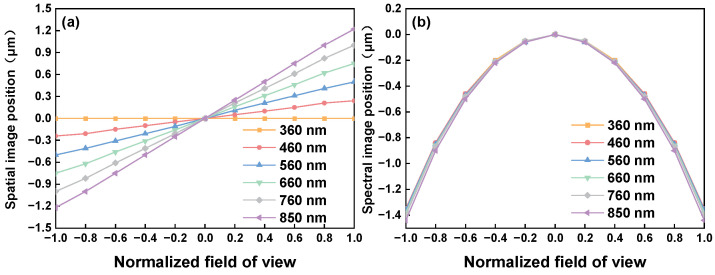
Smile curves (**a**) and keystone curves (**b**) of the spectrometer.

Another group of indicators demonstrating the performance of the imaging spectrometer is the smile and keystone. The curves are shown in Figure 8. The smile is less than 1.5 μm and the keystone is less than 1.25 μm, with both being smaller than the size of 0.1 pixels.

An analysis of the tolerance requirements to meet the imaging quality after processing and assembly was performed. The main tolerances (TOLs) are listed in Table 3.

**Figure 9:**
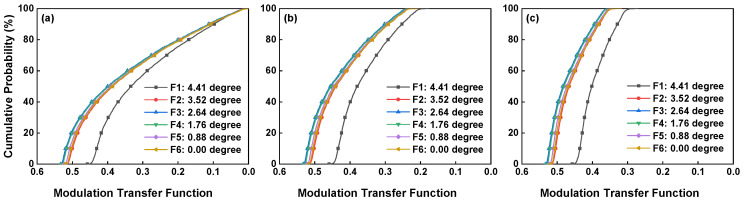
TOL MTF with grating surface RMS of 1/10 λ (**a**), 1/15 λ (**b**), and 1/20 λ (**c**).

The surface roughness of all the mirrors obtained from this analysis is 0.02 λ and the roughness of the grating is 0.1 λ. Since the MTF of the instrument can be expressed as the product of the optical system’s MTF and the detector’s MTF, a probabilistic analysis of the MTF under the influence of tolerances was conducted; by combining the detector’s MTF, the tolerance MTF values are 1/10 λ, 1/15 λ, and 1/20 λ for the RMS of the grating. The results are shown in Figure 9, which shows that when the TOL is frozen, the RMS needs to be greater than 1/15 λ to achieve a 60% probability of greater imaging quality. In this study, the grating is 1/10 λ; however, due to the small number of elements and the ease of finding the relative reference, the final assembly stage also achieved a relatively ideal image quality.

### 3.3. Stray Light Analysis

**Figure 10:**
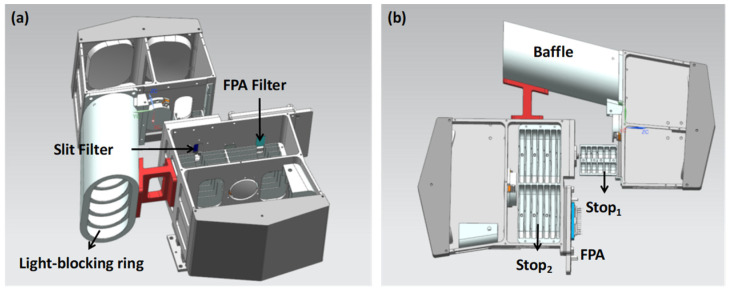
Structure of the instrument used to analyze the +2nd-order stray light. Positions of the filters in the structure (**a**). Positions of the light-blocking components within the instrument (**b**).

To analyze the stray light levels inside the instrument, a baffle, a light-blocking ring, and two other stops were installed. The internal mechanical structure is shown in Figure 10. Among these components, the baffle, light-blocking ring, stop 1, and stop 2 mainly block stray light within the FOV. The slit filter selects the working wavelength range, and the FPA filter primarily suppresses the +2nd- and 0th-order diffracted light from the grating.

**Figure 11:**
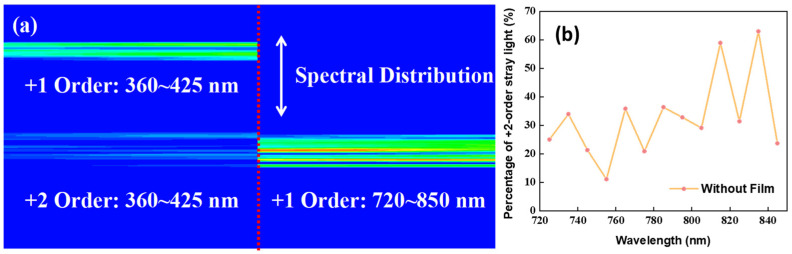
Spectrum position (**a**); comparison curve of imaging signals vs. non-imaging signals (**b**).

The introduced stray light in the optical system mainly includes the light outside the FOV, the reflection in the enclosure and gaps, and the spectral stray light from the grating. For a slit imaging spectrometer, the slit width and certain mechanical structures can effectively prevent the transmission of stray light from outside the scene. Combining the characteristics of the first three types of stray light, the use of extinction paint, light traps, hoods, and sealing gaps can effectively reduce the stray light to below 1%. Therefore, this study primarily focused on the analysis of multiple overlapping spectral stray light rays after grating diffraction by establishing a model and analyzing the distribution of wavelength energy from the detector. For spectral stray light, the +2nd-order diffraction of light from the grating will have the most significant impact. The stray light ratio was set as the SLR:

(15) $$SLR=\frac{Signal_{2}}{Signal_{1}}\cdot 100\%$$

where *Signal*_1_ represents the irradiance of the +1st-order diffraction light of the pixel, and *Signal*_2_ represents the +2nd order. The structure of the optical machine was modeled and the ratio of the imaged and non-imaged light was analyzed at the 720–850 nm band position. In this case, the stray light ratio under each spectral channel was analyzed separately using 10 nm as the sampling interval and then fitted by discrete points. Since the +2nd order is the most dominant stray light, only this part of the stray light was briefly analyzed. As shown in Figure 11, the +2nd-order stray light overlaps with the +1st-order light in the spectral direction on the image plane. Due to the light source being set like that of Earth’s reflectance curve, and combined with the system’s transmission efficiency, the proportion of stray light signals is higher in the longwave region than in the shortwave region, reaching 60% at the peak.

**Figure 12:**
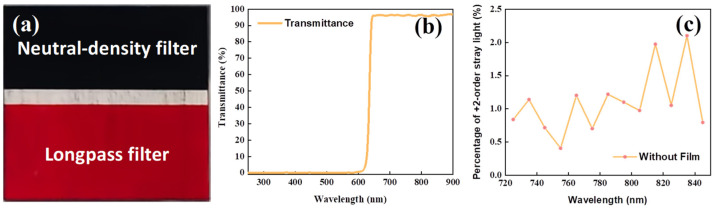
Window film (**a**); transmittance curve (**b**); stray light suppression effect curve (**c**).

Based on the above analysis, it is necessary to suppress the +2nd-order diffraction light from the shortwave positions in the longwave positions. A region-specific filter film was applied to the detector window as coating, which was divided into three areas: an absorption region, a transmission region, and a long-wavelength transmission region. The resulting suppression effect is shown in Figure 12, with the longwave position reaching below 0.2%. In this context, the long-wavelength transmission region of the detector window achieves a cutoff depth of OD4, equivalent to 10^−4^, thereby effectively suppressing the +2nd-order diffraction light.

### 3.4. Grating, Coating Mirrors, and Detector

#### 3.4.1. Manufacture of Grating

The diffraction efficiency of a convex grating in the 360–850 nm region needs to be higher than 50%, and a groove shape for the convex grating should be considered. The grating parameters used in this study are listed in Table 4. The diffraction energy of the +1st order is higher than 50%, and the groove density is 58 lines/mm.

**Figure 13:**
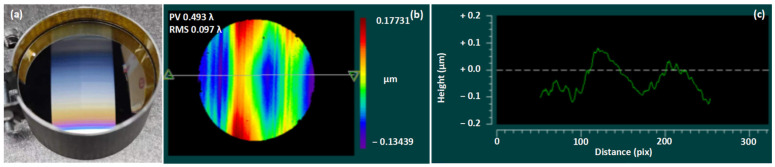
High diffraction efficiency of convex grating (**a**); surface RMS (**b**); surface profile (**c**).

Due to the low diffraction efficiency of holographic gratings, the manufacturing method of mechanical engraving was selected. The depth of the grating grooves is 0.32 μm with a blazed angle of 2.4 degrees after manufacturing. The wavefront quality of the grating surface measured with an interferometer is shown in Figure 13. The result shows that the RMS is 0.097 λ, constrained by the high efficiency of the grating.

**Figure 14:**
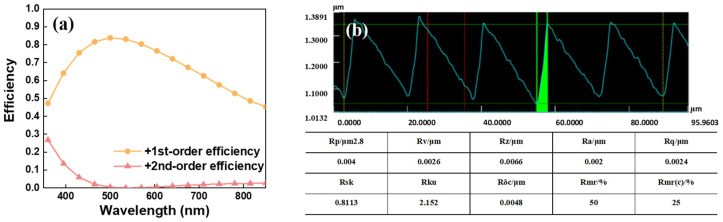
The diffraction efficiency curves (**a**); roughness testing with a laser microscope (**b**).

Since other orders are far from the +1st order in the imaging plane, only the diffraction efficiencies of the +1st and +2nd orders are listed in Figure 14 after measurement. The diffraction efficiency of this convex grating remains above 50% across the working wavelength range, reaching a peak of 83% at 530 nm. It shows a high energy of the +2nd-order diffraction near 400 nm. The profiles of the grooves observed under a laser microscope and the obtained surface roughness are also shown in Figure 13.

#### 3.4.2. Manufacture of Coated Mirrors

For the reflective mirrors, this study imposed some requirements, as shown in Table 5.

**Figure 15:**
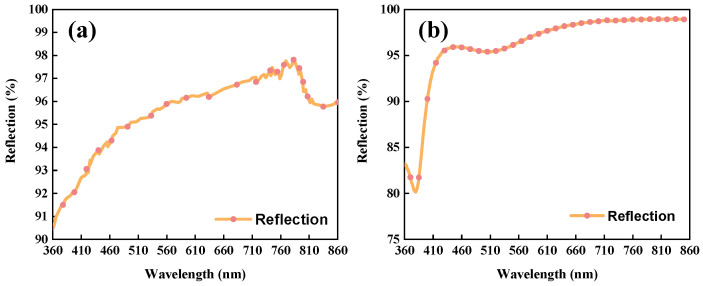
Efficiency of TMA mirrors (**a**); efficiency of Offner mirrors (**b**).

Due to the requirement for high reflection within the wavelength range, the mirror surfaces were coated with silver. The efficiencies of the mirrors in the TMA and Offner system are shown in Figure 15. All mirrors’ reflection is above 90% on average, but the Offner mirrors have a lower reflection within the 360–400 nm range. According to the efficiency transmission law of the system, the energy in the final imaging plane is lower in the 360–400 nm range, which decreases the SNR.

#### 3.4.3. Slit Filter and Detector Performance

**Figure 16:**
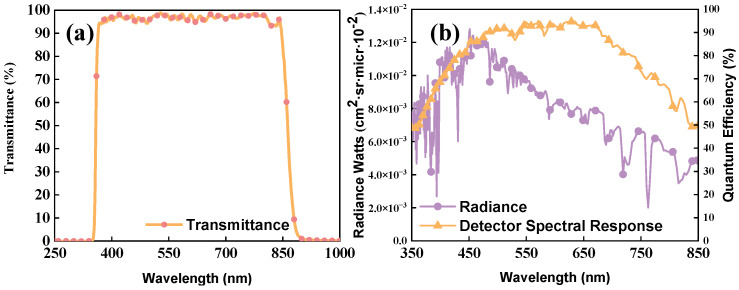
Slit filter transmission (**a**); total radiance and spectral response of detector (**b**).

Figure 16a shows the transmittance of the slit filter, whose transmission exceeds 94% in the range of 360–850 nm, with an outside waveband efficiency of less than 0.4% and a cut-off depth of OD3. There is a 5–8% decrease in efficiency at the edges of the waveband, resulting in a small impact on the energy of the spectral channels at 360–370 nm and 840–850 nm. For SOM detection, the surface albedo is 30% and the solar zenith angle is 30 during the window period; MODTRAN was used to describe the ground radiance of the working waveband, and the detector’s quantum efficiency is listed in Figure 16b. This study chose the back-illuminated CMOS GENSE400BSI; the efficiency is always above 50% in the 360–850 nm range and a peak of 92% is obtained at 580 nm.

## 4. Assembly and Tests

**Figure 17:**
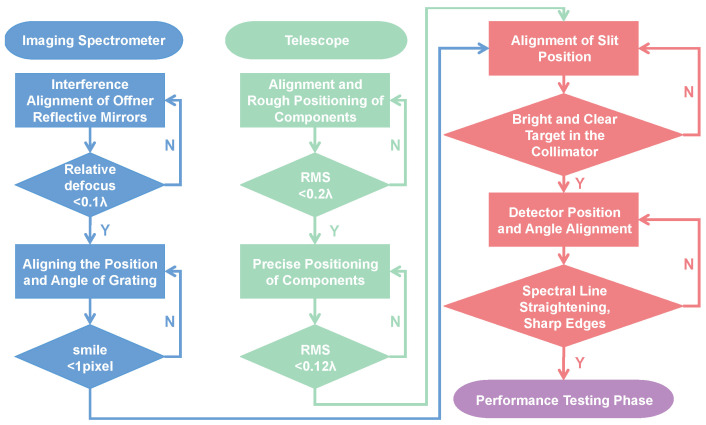
Whole machine integration process diagram.

The specific process is shown in Figure 17.

The assembly of the whole machine is divided into (1) assembling the two reflective mirrors of the spectrometer; (2) installing the slit, grating, and detector; (3) assembling the telescope; and (4) aiming the slit.

### 4.1. Assembling Imaging Spectrometer

**Figure 18:**
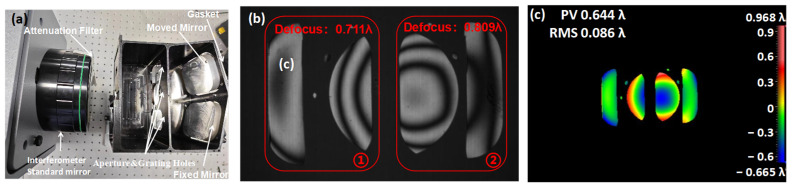
Optical path (**a**); interferogram (**b**); result of the assembled structure (**c**).

Because the two mirrors of the spectrometer are the same, an interferometer can be used to assist the assembly, such as in the surface shape test. The assembly and the results are shown in Figure 18.

This process used an interferometer with an attenuation filter to reduce the impact of the high reflection efficiency of the optical path system and improve the image contrast. The process was divided into two steps of coarse and fine adjustment. After fixing one of the mirrors, the other mirror was moved to find the angle information, and then the axial distance was adjusted through fine adjustment. The interference image was divided into two areas, where area 1 is a fixed mirror and area 2 is an active mirror. Because a standard mirror with an F number of 0.75 is enough to cover the two spherical mirrors to be measured, the interference image of the two mirrors should have coherence and the number of circular fringes is as small as possible. The defocus between the two mirrors is 0.09λ or 58 μm, which meets the tolerance requirements.

**Figure 19:**
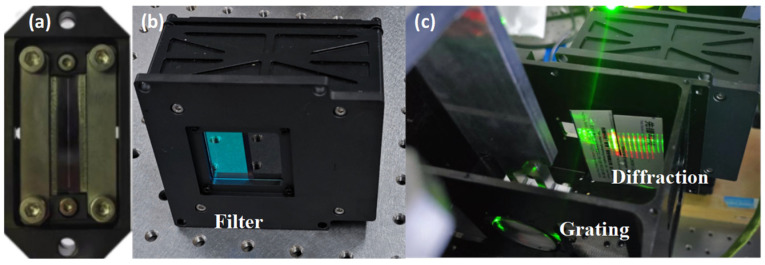
Slit (**a**); detector (**b**); and the positions of the grating and imaging plane (**c**).

After fixing the adjusted mirrors by cementing, the slit, grating, and detector were ready to be installed. The slit and detector are shown in Figure 19. The purpose of this step was to find the angle of rotation of the grating and the spatial position of the slit.

**Figure 20:**
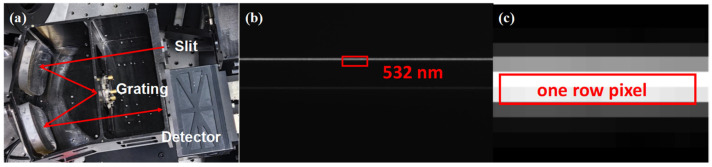
Optical path (**a**); spectral line in one row of pixels (**b**); and magnified view (**c**).

Because of the Offner imaging characteristics, the imaging plane is inclined when the slit is not parallel to the grating grooves. When the grating angle is wrong, the spectrum will bend towards the longwave or shortwave position. During assembly, the tilted and bent spectral lines were straightened until they were distributed within one row pixel, and the spectral lines at the center and edge fields were equally clear. The results are shown in Figure 20; the 532 nm spectral line has the same clarity in the full FOV and is stably distributed in one row pixel. The magnified view of the area of a single spectral line shows that the grayscale value of the central pixel is higher than the sum of the grayscale values of the other pixels, and the spectral line can be considered as distributing in one row of pixels (2 × 11 μm).

**Figure 21:**
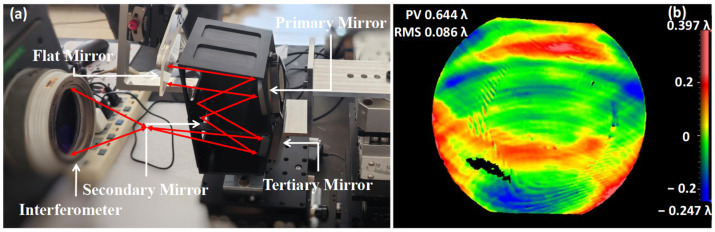
Optical path (**a**); the results of fine adjustment (**b**).

The telescope uses a self-aligning adjustment method. Due to the silver coating on the three mirrors, the only requirement for the flat mirror is 1/40λ in roughness, and coating is not necessary, to ensure a high level of fringe contrast. Initially, the secondary mirror was fixed, and the primary and tertiary mirrors were displaced using a displacement table to align the focus of the telescope with the interferometer’s focus. At this step, the RMS of the wave aberration of coarse adjustment was less than 0.2λ. Then, fine adjustment of the mirrors’ angle was performed to achieve a wave aberration of 0.1λ, thereby approaching the wave aberration of the design. The optical path of installing and adjusting and the results of fine adjustment are shown in Figure 21.

**Figure 22:**
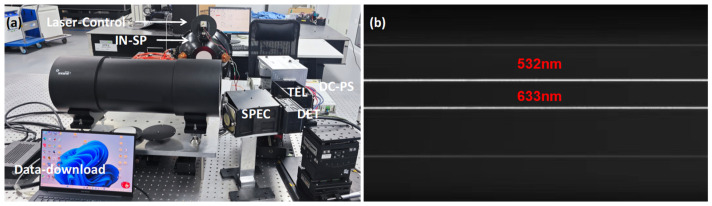
Assembling scene (**a**); spectral imaging (**b**).

After the above steps, the spectrometer and telescope components both exhibit great imaging quality. The final assembly process was aligning the spectrometer with the telescope at the slit position by finding an appropriate relative angle and position between the two components. The detector was moved to compensate for the defocus, and the spectral lines were tilted and bent. The process used a Laser Control System (Laser-Control), an integrating sphere (IN-SP), and a collimator to provide an incident condition with a single spectrum. The optical equipment and alignment scene are shown in Figure 22. At last, several spectral lines fall within their own one row of pixels with clear edges. 

### 4.2. Lab Test

To verify the performance of the whole instrument after assembly, the instrument was tested in the laboratory. This included an SNR test for simulated ground-reflected irradiance in the waveband, a spectral resolution test, and a focal length and MTF test.

#### 4.2.1. Focal and Spatial Resolution

**Figure 23:**
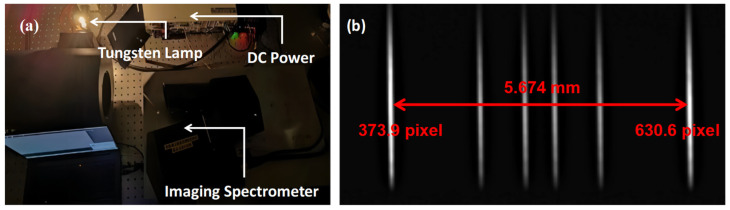
Testing path (**a**); detector image (**b**).

In the laboratory, the equipment used for focal length testing included a collimator, a tungsten lamp, and a target plane. The testing path and the detector image are shown in Figure 23. By using known spacing of the engraved lines, the pixel spacing on the image plane, and the collimator focal length, the focal length of the instrument can be calculated.

**Figure 24:**
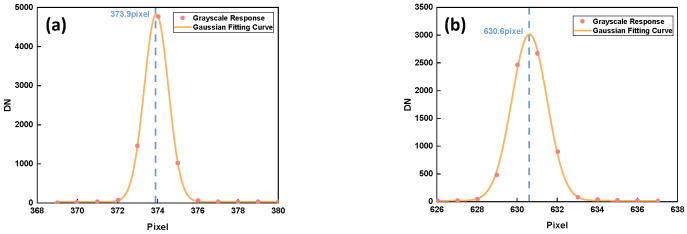
Grayscale response and Gaussian fitting curves of pixels: left edge (**a**); right edge (**b**).

The imaging position of line pairs with an actual spacing of 5.674 mm was analyzed. To accurately calculate the position of each line, the gray analysis and Gaussian fitting of the imaging position were conducted to obtain the position information of 0.1 pixels; the results are shown in Figure 24.

**Figure 25:**
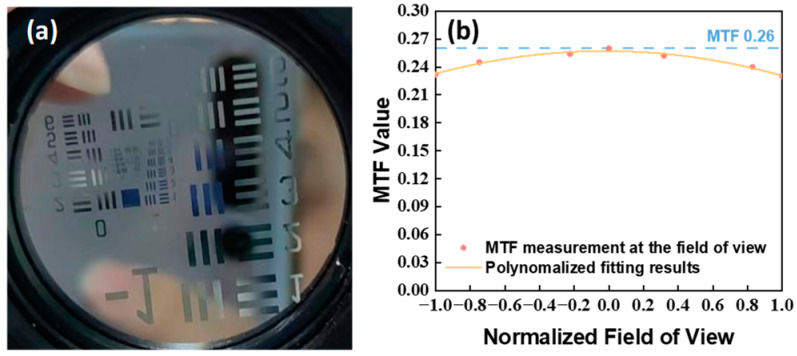
The 1951 USAF resolution test plane (**a**); MTF testing and fitting curve (**b**).

The results show that when the lines’ spacing is 5.674 mm, the pixel span is 256.7, the focal length is 141.71 mm, and the spatial resolution is 100.6 m. When the target was changed to the 1951 USAF resolution test plane, the 5-2 line pairs were used to match the frequency of the instrument’s detector. The random point test was performed at the center and edge of the FOV, and MTF curve fitting was carried out on the normalized FOV; the results are shown in Figure 25.

The MTF is >0.26 in the center and >0.23 at the edge FOV at the Nyquist frequency, which meet the detection requirements.

#### 4.2.2. Spectral Response and Resolution

**Figure 26:**
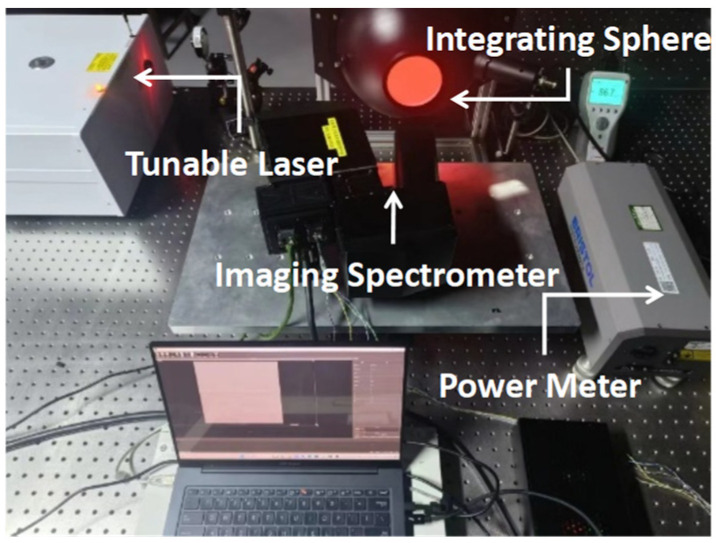
Test of spectral response and resolution.

The spectral performance of the instrument was tested by measuring the first and last image positions in the spectral dimension, determining the range of the bands, and sweeping the spectrum with a tunable laser; the testing path is shown in Figure 26.

**Figure 27:**
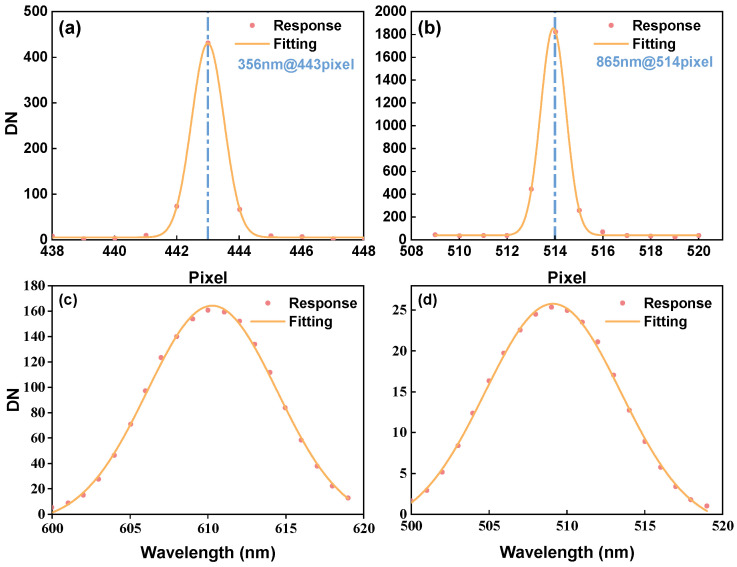
The test result of response at 356 nm (**a**). The test result of response at 865 nm (**b**). The test result of FWHM at 510 nm (**c**). The test result of FWHM at 610 nm (**d**).

According to the test results, the spectral response range of the instrument is 356–865 nm, including a range requirement of 360–850 nm. The scans exhibited a 20 nm bandwidth with 510 nm and 610 nm as the center wavelengths in a step of 1 nm, and the grayscale changes of the pixels of the center wavelengths were recorded. Finally, the spectral response curves were fitted and the full width at half maximum (FWHM) was solved. The curves are shown in Figure 27.

The FWHM of the two wavelength points at 510 nm and 610 nm is 9.557 nm and 9.412 nm, respectively, which can be regarded as a full-spectrum spectral resolution of less than 10 nm due to the high linearity of the dispersion of the grating spectrometer.

#### 4.2.3. SNR

**Figure 28:**
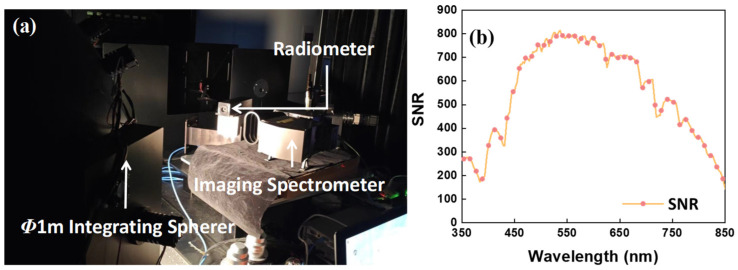
Test equipment (**a**); SNR curve (**b**).

In this step, ground-reflected irradiance was simulated using a light source and an integrating sphere. However, due to the lower brightness of the light source in the 360–400 nm band compared to other bands, the simulation of ground-reflected irradiance at the same input power faced challenges. To overcome this, the input power and integration time were increased to simulate ground-reflected irradiance at shortwave positions. During this test, the SNR was first measured in the 400–850 nm band. Then, the power of the light source was increased to enhance the signal in the shortwave band for SNR measurements at those positions. Notably, only the light source power was adjusted during the test, while the integration time was kept at 13 ms. This setup was consistent with a ground resolution of 100 m and an equivalent ground line flight speed of 7.8 km/s, resulting in a maximum effective exposure time of 13 ms for each ground spatial point, thus ensuring spatial data continuity. The results show that at shortwave positions (360–400 nm), the SNR reached 200, and the value reached 800 at 550 nm. Detailed information about the testing equipment and the results are provided in Figure 28.

## 5. Discussion

As can be seen from Equation (2), the stronger the spectral resolution of the spectrometer (the narrower the wavelength range corresponding to a single pixel), the lower this portion of energy will be. The ground resolution *s* is related to the ground scan speed *v* at orbital altitude, corresponding to the maximum integration time *t* of the instrument in normal operation, such that *s* = *vt*. The SNR comparison in this article refers to the typical operating conditions (corresponding to its own ground and spectral resolution) of each instrument when observing soil. Table 6 shows the resolution indicators of some devices.

**Figure 29:**
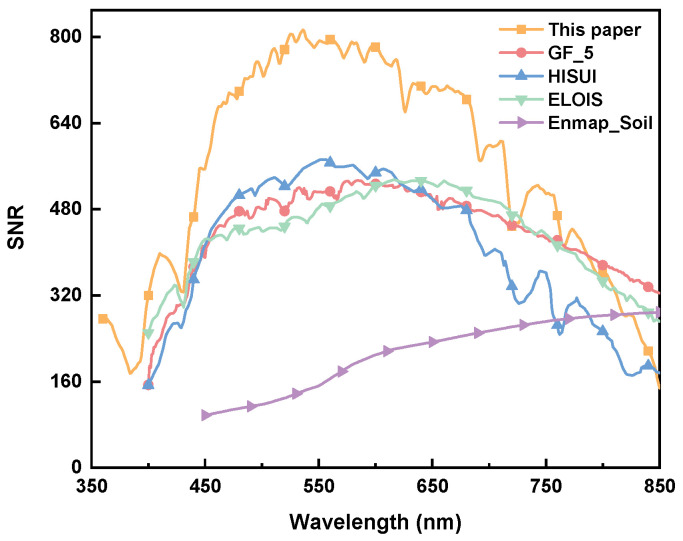
The trend curves of SNR for each instrument.

However, since each instrument has different primary observation targets, the energy of the observation target corresponds to different solar elevation angles and ground albedos. To unify the observation objectives, the target energy information is standardized to the energy emitted at a solar zenith angle of 30° and a ground albedo of 0.3, allowing for a comparison of the SNR that each instrument can obtain under these conditions. Figure 29 primarily assesses the overall SNR that the instruments can achieve when observing soil organic matter (SOM) targets.

However, it can also be observed that the curve has lower values than 200 in the 370–390 nm and 840–850 nm channels. The reason for this phenomenon is the lower mirror reflectivity in the shortwave range, leading to the higher energy decay of the central wavelength after multiple reflections. In particular, the shape of the shortwave valley is very similar to the valley in Figure 15b. In the longwave range, it is mainly due to the low reflective irradiance of the ground and the slight decrease in efficiency in the working band at the edge of the slit filter. According to the MTF test, the center of the FOV reached 0.26, and with a surface quality of the grating of 0.097 λ, the assembly probability reached 60%. As shown as Figure 9, when the wavefront error RMS on the grating surface reaches 1/15λ or higher, the instrument can achieve a superior imaging ability under the same assembly probability.

Therefore, when weak targets are detected, subsequent designs need to consider expanding the working band of the filter to meet the in-band transmission efficiency. Simultaneously, it is important to control the mirrors’ surface coating to achieve a 90% reflectivity at around 350 nm. As for the high-efficiency diffraction grating, a higher surface quality is required to improve the efficiency. These improvements will enable similar instruments to achieve higher performance.

## 6. Conclusions

This paper discusses the design of an imaging spectrometer with a high SNR, which was developed based on the basic structures of TMA and Offner for SOM detection. The optical design has an F number of three and is capable of achieving a resolution of 100 m and a swath of 100 km in a 648 km orbit, with good imaging quality. In the optical link, silver-coated mirrors, a back-illuminated CMOS detector, and a high-efficiency grating under wide band are used to improve the energy transmission efficiency, obtaining an SNR higher than 200 within the 360–850 nm range, with the peak reaching 800. The simulation analysis shows that the stray light of the +2nd-order diffraction in the spectral dimension is suppressed to the level of 0.2%. Additionally, the smile and keystone of the spectrometer are less than 0.1 pixels. The optical components were progressively integrated and tested in the laboratory, with the final tests indicating that the central field’s MTF is better than 0.26 at the Nyquist frequency, the edge field’s MTF is better than 0.23, and the spectral dimension resolution is better than 10 nm, which can meet the detection requirements of SOM. At the same time, to facilitate subsequent research work, this paper discusses the key technical challenges. The achievement of a high SNR provides a technical basis for the application of such an Offner spectrometer in remote sensing in the weak-signal space.

## Figures and Tables

**Table 1:** The specifications of the instrument.

Parameter	Index
Spectral resolution	10 nm at 0.36–0.85 μm
GSD	100 m
Pixel size	22 μm × 22 μm
F number	3
Numerical aperture (NA)	0.1667
Focal length	141.42 mm
Entrance pupil diameter	47.14 mm
Swath at orbit	100 km at 648.2 km
FOV angle	±4.41°
Slit length	21.8 mm

**Table 2:** The parameters of the surfaces.

Surface	Component	Radius/mm	Thickness/mm	k
1	Primary Mirror	−388.20	80.02	−3.671
2	Secondary Mirror	−155.94	80.02	/
3	Tertiary Mirror	−164.47	138.17	0.182
4	Filter 1: Front	infinity	0.50	/
5	Filter 1: Rear	infinity	2.00	/
6	Slit	infinity	2.00	/
7	Filter 2: Front	infinity	0.50	/
8	Filter 2: Rear	infinity	157.43	/
9	Offner Mirror 1	156.90	75.82	/
10	Offner Grating	78.29	75.82	/
11	Offner Mirror 3	156.90	157.74	/
12	Window Filter: Front	infinity	1.00	/
13	Window Filter: Rear	infinity	0.615	/
14	Image Surface	infinity	0	/

**Table 3:** The tolerance of the surfaces.

Component	Radius TOL (mm)	Thickness TOL (mm)	Eccentricity TOL (mm)	Tilt TOL (′)	Wedge TOL (′)
Primary Mirror	0.08	0.1	0.04	0.3	/
Secondary Mirror	0.08	0.3	0.08	0.3	/
Tertiary Mirror	0.08	0.5	0.02	1	/
Filter 1	/	0.5	/	0.3	11.4
Filter 2	/	0.5	/	0.3	11.4
Offner Mirror 1	0.08	0.06	0.02	1	/
Offner Grating	0.1	0.06	0.1	0.3	/
Offner Mirror 3	0.1	0.5	0.02	1	/
Window Filter	/	0.5	/	0.3	10.9

**Table 4:** The parameters of grating.

Parameter	Value	Parameter	Value
Wave band	0.36–0.85 μm	Groove density	58 lines/mm
Kind	Blazed	Coating	Aluminum
Radius	78.28 mm	RMS	0.096λ
Efficiency	>50%	Work order	+1

**Table 5:** The parameters of mirrors.

Parameter	Value
Wave band	0.36–0.85 μm
Efficiency	>95% at 550 nm
Coating	Silver
RMS	0.02λ

**Table 6:** The parameters of instruments.

Instruments	Ground Resolution	Spectral Resolution
GF5	30 m	5 nm
HISUI	20 m	10 nm
ELOIS	35 m	2.5 nm
ENMAP	30 m	6.5 nm
This paper	100 m	10 nm

## Data Availability

The data that support the findings of this study are available from the corresponding author upon reasonable request.
